# Supplementary material for: Pluronic gel-based burrowing assay for rapid assessment of neuromuscular health in C. elegans
Source: Sci Rep. 2019 Oct 23;9:15246. doi: 10.1038/s41598-019-51608-9 (PMC6811592; doi:10.1038/s41598-019-51608-9)
Supplement: Supplementary file 1 — Supplementary information [file 41598_2019_51608_MOESM1_ESM.docx]

**Supplementary information**

**Pluronic gel-based burrowing assay for rapid assessment of neuromuscular health in *C. elegans***

Leila Lesanpezeshki^1^, Jennifer E. Hewitt^1,3^, Ricardo Laranjeiro^2^, Adam Antebi^3^, Monica Driscoll^2^, Nathaniel J. Szewczyk^4^, Jerzy Blawzdziewicz^5^, Carla M.R. Lacerda^1^ and Siva A. Vanapalli^1*^

*^1^Department Of Chemical Engineering, Texas Tech University, Lubbock, TX, USA*

*^2^Department of Molecular Biology and Biochemistry, Rutgers, The State University of New Jersey, Piscataway, NJ, USA*

*^3^Department of Molecular Genetics of Ageing, Max Planck Institute for Biology of Ageing, and Cologne Excellence Cluster on Cellular Stress Responses in Aging-Associated Diseases (CECAD), University of Cologne, Cologne, Germany*

*^4^MRC/Arthritis Research UK Centre for Musculoskeletal Ageing Research, University of Nottingham, United Kingdom & National Institute for Health Research Nottingham Biomedical Research Centre, Derby, UK*

*^5^Department of Mechanical Engineering, Texas Tech University, Lubbock, TX, USA*

Supplementary Video S1. 2-hr time lapse of burrowing assay.

Supplementary Video S2. Pmyo-3 GCaMP3.35 burrowing in 26 % w/w PF-127.

Supplementary Note S1. The effect of Pluronic and burrowing environment on stress response

Supplementary Figure S1. Testing the effect of gravity on burrowing in 26 % w/w Pluronic

Supplementary Figure S2. Burrowing assay in reverse

Supplementary Figure S3. The stress response of TJ356 in Pluronic

Supplementary Figure S4. Gel elastic modulus and yield stress as a function of Pluronic concentration

Supplementary Figure S5. Characterization of burrowing behavior in 26 % w/w PF-127

Supplementary Figure S6. Additional muscle-specific gene expression in fast burrowers compared to slow burrowers

Supplementary Table S1. Dense body and M-line mutants used to study the muscle defects

Supplementary Table S2. List of genes tested for qPCR

Supplementary Table S3. List of primers used for qPCR

Supplementary Table S4. Cycle threshold values for the reference genes used to calculate the relative gene expression

**Supplementary Video S1. 2-hr time lapse of burrowing assay.** Wild-type animals are burrowing toward the attractant (*E. coli*) on the top surface in 26 % w/w PF-127 within the assay duration of 2 hours.

**Supplementary Video S2. Pmyo-3 GCaMP3.35 burrowing in 26 % w/w PF-127.** As muscles actuate during burrowing, they appear brighter due to an elevation in calcium ion levels. In the beginning, the animal has acquired a 3D posture, so not all the body appears in the same focal plane. As it moves forward, the tail is emerging in the same focal plane of the microscope, making all the animal’s body become in focus.


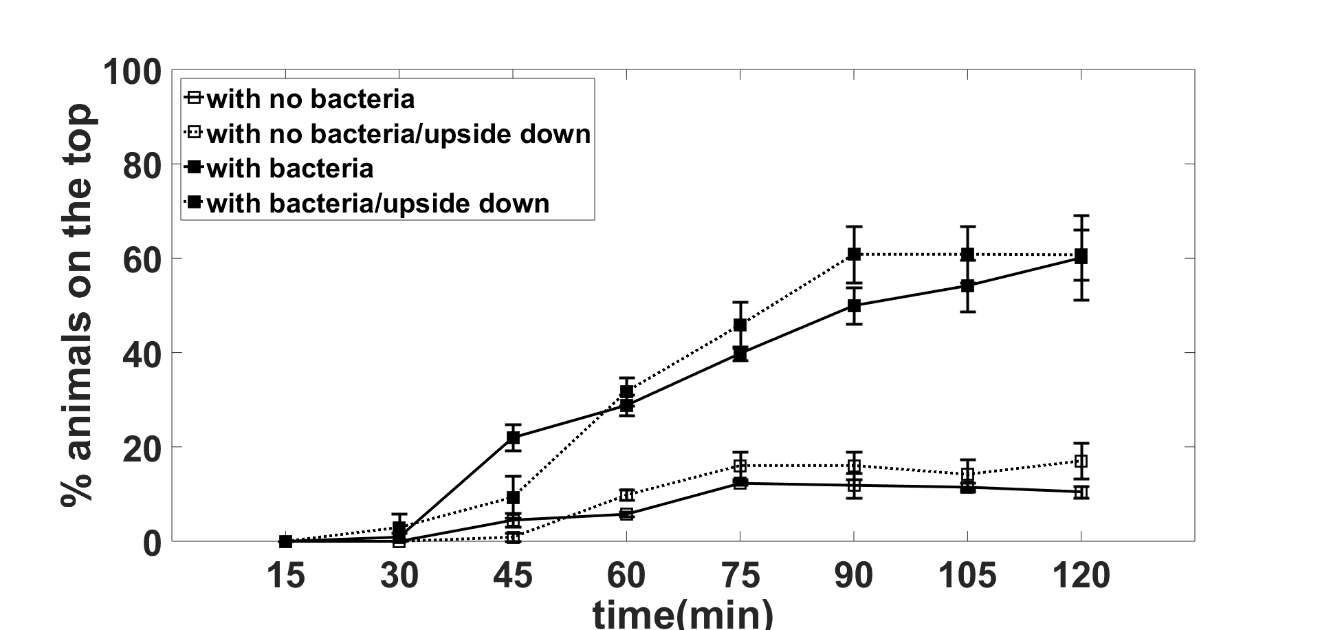


**Figure S1. Testing the effect of gravity on burrowing in 26 % w/w Pluronic**. Gravity did not show any significant effect on the burrowing rate of wild-type animals. Gel thickness, H = 0.9 cm (N= 37 on average. 3 replicates per condition).


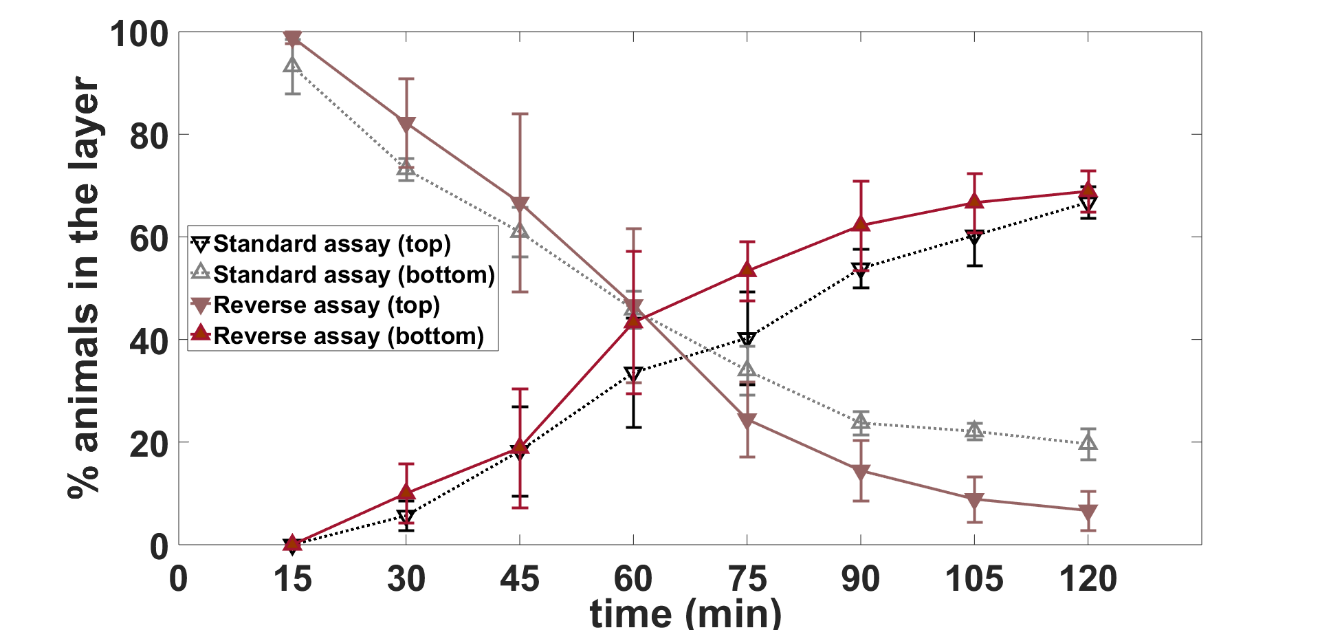


**Figure S2. Burrowing assay in reverse**. In the reverse setup, the attractant (100 μL bacteria) was loaded on the bottom of the well plate and then fixed with a thin layer of Pluronic to prevent mixing of the attractant with the main Pluronic layer on top. Next, the top layer of Pluronic (~0.75 cm) was cast above the attractant layer, and then the animals were added to the very top surface. Since the animal crawling trails on the surface of the Pluronic produced a shadow, which can visually interfere with inspecting the bottom layers, a thin layer of Pluronic (~1 mm) was added to the top, so that they were not crawling on the surface anymore H = 0.75 cm (N=30 animals with 3 replicates).

The standard assay is the same as described (see Methods, Pluronic-based burrowing assay), except that 100 μL of *E. coli* was used as the attractant to match the reversed burrowing assay. H = 0.75 cm (N=33 animals with 3 replicates).

**Supplementary Note S1. The effect of Pluronic and burrowing environment on stress response**

To clarify the response of *C. elegans* to long duration exposure to PF-127, we evaluated stress induction in both the Pluronic liquid and gel environments. We used the TJ356 and studied the stress response under four different conditions of: (i) NGM plate with food (negative control), (ii) NGM plate without food (starvation), (iii) swimming animals in 16 % w/w PF-127 (liquid environment), and (iv) burrowing animals in 26 % w/w PF-127 (gel environment) without food. Day 1 adult animals kept at 37 ˚C for an hour served as the positive control. 30 animals were imaged at time points of 1 and 2.5 hours and the percentage of animals showing nuclear localization of DAF-16::GFP was assessed. The experimental procedure was as follows: The age synchronized TJ356 animals were cultured on NGM plates until they reached day 1 of adulthood. Animals were then transferred to: NGM plates seeded with food (i), unseeded NGM plates (ii), 3 mL of 16 % w/w PF-127 in a 12 well plate to serve as a swimming environment for the animals (iii), and 1 mL of 26 % w/w Pluronic on a glass slide and sandwiched with a coverslip on top (iv) (See Methods, Behavioral analysis during burrowing). At each time point, 30 animals from each condition, except (iv) were transferred to 10 μL of 26 % w/w Pluronic on a glass slide compacted using a coverslip to slow down the animals’ movement to prepare them for imaging. As the animals in (iv) were already sandwiched on a glass slide, there was no need to transfer them to another glass slide, and the coverslip was squeezed more to confine the animals and restrict their movements. 30 animals were imaged in each condition and time point with one replicate.

We observed that the at the 1-hour time point, about 27% of the animals in the burrowing/gel environment showed a stress response. This is higher than the 10% and 3% of the animals in the swimming/liquid and plate/starved conditions, respectively. None of the animals in the standard NGM plate condition with food showed any stress response. A more pronounced effect is observed at 2.5 hours but with a similar trend. Thus, for the 2-hour duration of the burrowing assay, it appears that the mechanical resistance of the Pluronic gel environment induces a greater stress response.


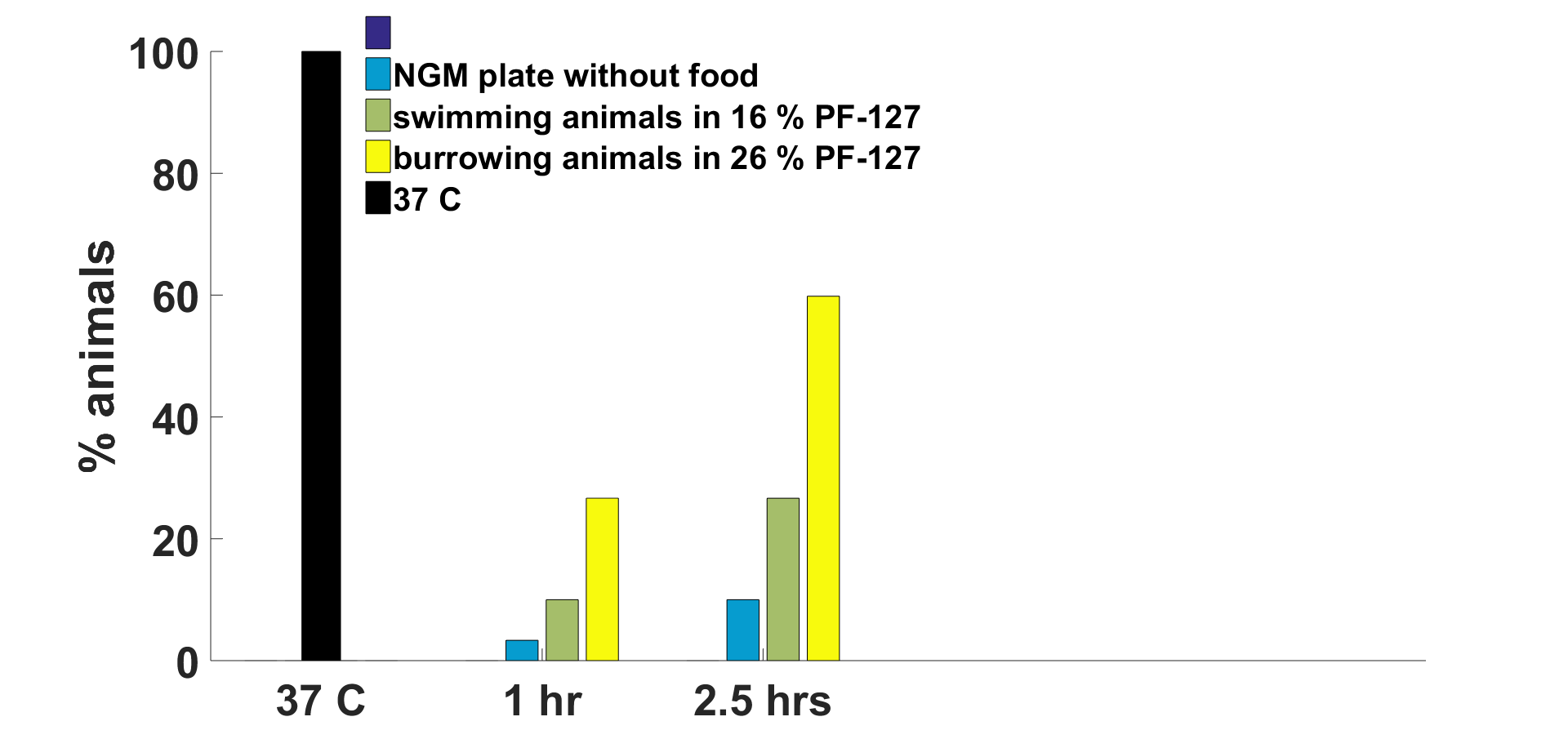


**Figure S3.** **The stress response of TJ356 in Pluronic**. (left) Stress response was measured by nuclear localization of DAF-16::GFP in TJ356 strain. (right) Percentage of the TJ356 animals showing DAF-16::GFP nuclear localization, primarily in intestinal cells, on NGM plates without food, swimming in 16 % w/w Pluronic, and burrowing in 26 % w/w Pluronic. None of the animals in the standard NGM plate condition with food showed any stress response.

| Pluronic concentration (% w/w) | Yield stress (Pa) |
| --- | --- |
| 24 | 251.2 |
| 26 | 316.2 |
| 28 | 398.1 |
| 30 | 501.2 |


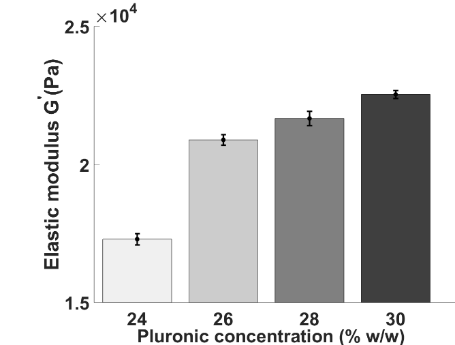


**Figure S4. Gel elastic modulus and yield stress as a function of Pluronic concentration**.

The elastic modulus for 24 % w/w 26 % w/w, 28 % w/w and 30 % w/w are 17.3 kPa, 20.9 kPa, 21.7 kPa and 22.5 kPa, respectively. The yield stress for 24 % w/w to 30 % w/w varies from 251.2 Pa to 501.2 Pa.

Rheology measurements were performed using an AR2000 rheometer with parallel plate geometry (40 mm diameter and 500 μm gap). The base plate was initially set at 10 ˚C and equilibrated at 20 ˚C for doing all the measurements. Samples were kept at 4 ˚C prior to the experiment. Oscillatory shear stress sweep from 1 to 1000 Pa at the frequency of 1 Hz was used to find the linear viscoelastic region. The yield stress was determined as the critical oscillatory stress when the elastic modulus deviated more than 10% from the linear viscoelastic region.


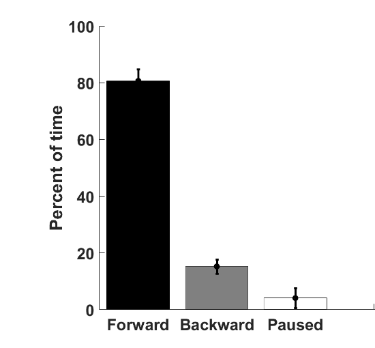


**Figure S5. Characterization of burrowing behavior in 26 % w/w PF-127.** Percent of time duration wild-type animals spent in each phase of locomotion. Animals spent most of their time moving forward.

| **Gene** | **Human ortholog or homolog** | **Description (From WormBase WS264)** |
| --- | --- | --- |
| *uig-1* | PLEKHG1, PLEKHG3 | Localized to the striated muscle dense body. Expressed in the head, the muscle cell, and the tail. |
| *unc-82* | NUAK1, NUAK2 | Localized to the M band. Expressed in the body wall musculature. |


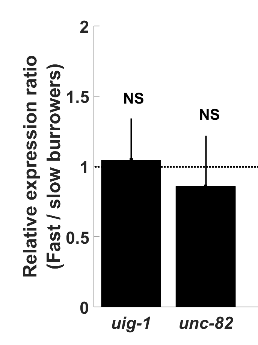


**Figure S6. Additional muscle-specific gene expression in fast burrowers compared to slow burrowers.**

No significant difference was detected for the expression of *uig-1* and *unc-82* for fast burrowers compared with slower ones*.* The same experimental methods as described before were utilized here. Experiments were conducted with Day 1 adults. N = 30 animals with three replicates. Error bars are standard deviation. NS>0.05

**Table S1. Dense body and M-line mutants used to study the muscle defects**

| **Mutant** | **Human homolog/ ortholog^1^** | **Phenotype** |
| --- | --- | --- |
| *dyc-1(cx32)* | Capon | Hyperactivity, overbent^2^ |
| *uig-1(ok884)* | PLEKHG1, PLEKHG3 | Normal swimming frequency, higher crawling frequency, higher minimum radius of curvature^3^, normal bending^4^ |
| *pfn-3(tm1362)* | Profilin | Slightly decreased thrashing^5^, bending-defective^4^ |
| *atn-1(ok84)* | α-actinin | Bending-defective^4^, abnormal dense bodies, normal thrashing^6^, higher minimum radius of curvature, normal crawling^3^ |
| *zyx-1(gk190)* | Zyxin | Bending-defective^4^ |
| *tln-1(e259)* | Talin | Unc phenotype^7^, exaggerated body bends (www.wormbase.org) |
| *unc-95(ok893)* |  | Movement defect^8^, bending-defective^4^, disrupted dense body and M-line^9^ |

**Table S2.** **List of genes tested for qPCR**

| **Gene** | **Human ortholog or homolog** | **Description (From WormBase WS264)** |
| --- | --- | --- |
| *unc-54* | Muscle myosin heavy chain (MHC B) | Required for locomotion and egg-laying a thick filament component that is expressed in multiple muscle cell classes |
| *tnt-2* | Troponin T | Expressed in the anal depressor muscle, reproductive system, and the body wall musculature. |
| *zig-12* | Titin | Localized to the endoplasmic reticulum, the sarcomere and the striated muscle dense body |
| *unc-27* | Troponin I | Required for coordinated motility, normal muscle morphology and proper sarcomeric organization |
| *unc-87* | Calponin like | Maintains the structure of myofilaments in body wall muscle cells |
| *unc-15* | Paramyosin | Physically interacts with MHC A, one isoform of myosin heavy chain (MHC) in striated muscle |
| *myo-3* | Myosin heavy chain | Essential for thick filament formation, and for viability, movement, and embryonic elongation; expressed in body muscle |
| *lev-11* | Tropomyosin 1 | An actin-binding contractile structural protein required for embryonic development, normal body morphology, and locomotion |
| *mup-2* | Troponin T | Affects embryonic body wall muscle cell contraction, sarcomere organization, cell positioning, and regulated muscle contraction in larval and adult body wall muscle |
| *mlc-1* | Muscle regulatory myosin light chain | Functions in the pharyngeal and body-wall muscle development, affects locomotion and growth; expressed in the body-wall muscles, pharyngeal muscles, and vulval muscles |

**Table S3. List of primers used for qPCR** (*cdc-42* and Y45F10D.4 are the control references)

| **Gene** | **Forward primer (5'-3')** | **Reverse primer (5'-3')** | **Amplification**  **efficiency** |
| --- | --- | --- | --- |
| ***cdc-42*** | CTGCTGGACAGGAAGATTACG | CTCGGACATTCTCGAATGAAG | 1.03 |
| ***lev-11*** | CCGCTGAAGAGAAAGTCCGT | TCGTCTCCGGTCTGAGTCAT | 0.93 |
| ***mlc-1*** | TGGAGCCTTTGCCATGTTC | CTTGACCTCATCCTCGTCCAAT | 0.99 |
| ***mup-2*** | AACGCAAGGCTAAGGCTGAT | AGCTCCGGCTTCAACTCTTC | 0.92 |
| ***myo-3*** | AGGGAGACTTGAAGGTTGCG | AGCGAGCTTAGCATTGGTGT | 0.93 |
| ***tnt-2*** | ATGGGGACGCAAAGAGAACG | AATTGGGTTGACTGGTGGCT | 1.00 |
| ***unc-15*** | CGAGGAAGCCAATGGACGTA | AAATCAGCTTGAGCGGTGGA | 0.95 |
| ***unc-27*** | CGTGGAAAGTTCGTCAAGCC | TCTTGAGGTTGGCACGGAAG | 0.98 |
| ***unc-54*** | ACTACCAACACGAAGCCGAG | GGCGTTAGCCTTGGAGAGTT | 0.94 |
| ***unc-87*** | ATGACTGGATTCGGACAGCC | AGCTTGAGAAGCAAAACGGT | 0.98 |
| **Y45F10D.4** | GTCGCTTCAAATCAGTTCAGC | GTTCTTGTCAAGTGATCCGACA | 0.94 |
| ***zig-12*** | GATCAGAGAACGGGTCGGTG | CTCCTCAAGCTCGTCTGGTC | 1.02 |

**Table S4. Cycle threshold values for the reference genes used to calculate the relative gene expression**

|  | *cdc-42* | Y45F10D.4 |
| --- | --- | --- |
| Fast 1 | 19.58432 | 19.94066 |
| Fast 2 | 19.14383 | 19.68750 |
| Fast 3 | 18.90726 | 19.66584 |
| Slow 1 | 18.78636 | 19.38099 |
| Slow 2 | 18.72359 | 19.41477 |
| Slow 3 | 18.60576 | 19.24362 |
| Average Ct values | | |
| Fast | 19.2118 | 19.76467 |
| Slow | 18.70524 | 19.34646 |

**References**

1 Gieseler, K., Qadota, H. & Benian, G. Development, structure, and maintenance of *C. elegans* body wall muscle. *WormBook: the online review of C. elegans biology*, 1 (2016).

2 Lecroisey, C. *et al.* DYC-1, a protein functionally linked to dystrophin in *Caenorhabditis elegans* is associated with the dense body, where it interacts with the muscle LIM domain protein ZYX-1. *Molecular biology of the cell* **19**, 785-796 (2008).

3 Hwang, H. *et al.* Muscle contraction phenotypic analysis enabled by optogenetics reveals functional relationships of sarcomere components in *Caenorhabditis elegans*. *Scientific reports* **6**, 19900 (2016).

4 Nahabedian, J. F., Qadota, H., Stirman, J. N., Lu, H. & Benian, G. M. Bending amplitude–A new quantitative assay of *C. elegans* locomotion: Identification of phenotypes for mutants in genes encoding muscle focal adhesion components. *Methods* **56**, 95-102 (2012).

5 Polet, D. *et al.* *Caenorhabditis elegans* expresses three functional profilins in a tissue‐specific manner. *Cell motility and the cytoskeleton* **63**, 14-28 (2006).

6 Moulder, G. L. *et al.* α-Actinin is required for the proper assembly of Z-disk/focal-adhesion-like structures and for efficient locomotion in *Caenorhabditis elegans*. *Journal of molecular biology* **403**, 516-528 (2010).

7 Brenner, S. The genetics of *Caenorhabditis elegans*. *Genetics* **77**, 71-94 (1974).

8 Zengel, J. M. & Epstein, H. F. Identification of genetic elements associated with muscle structure in the nematode *Caenorhabditis elegans*. *Cell motility* **1**, 73-97 (1980).

9 Broday, L. *et al.* The LIM domain protein UNC-95 is required for the assembly of muscle attachment structures and is regulated by the RING finger protein RNF-5 in *C. elegans*. *The Journal of cell biology* **165**, 857-867 (2004).
